# Association of Mast Cell-Derived VEGF and Proteases in Dengue Shock Syndrome

**DOI:** 10.1371/journal.pntd.0001505

**Published:** 2012-02-21

**Authors:** Takahisa Furuta, Lyre Anni Murao, Nguyen Thi Phuong Lan, Nguyen Tien Huy, Vu Thi Que Huong, Tran Thi Thuy, Vo Dinh Tham, Cao Thi Phi Nga, Tran Thi Ngoc Ha, Yasukazu Ohmoto, Mihoko Kikuchi, Kouichi Morita, Michio Yasunami, Kenji Hirayama, Naohiro Watanabe

**Affiliations:** 1 Division of Infectious Genetics, Institute of Medical Science, University of Tokyo, Tokyo, Japan; 2 Department of Virology, Institute of Tropical Medicine (NEKKEN), Nagasaki University, Nagasaki, Japan; 3 Arbovirus Laboratory, Pasteur Institute, Ho Chi Minh City, Viet Nam; 4 Department of Immunogenetics, Institute of Tropical Medicine (NEKKEN), Nagasaki University, Nagasaki, Japan; 5 Children's Hospital No. 2, Ho Chi Minh City, Viet Nam; 6 Center for Preventive Medicine, Vinh Long, Viet Nam; 7 Free Radical Research Institute, Otsuka Pharmaceutical Co., Ltd., Tokushima, Japan; 8 Center of International Collaborative Research, Nagasaki University, Nagasaki, Japan; 9 Global COE Program, Nagasaki University, Nagasaki, Japan; 10 Department of Tropical Medicine, Jikei University School of Medicine, Tokyo, Japan; Tropical Medicine Institute Pedro Kourí (IPK), Cuba

## Abstract

**Background:**

Recent *in-vitro* studies have suggested that mast cells are involved in Dengue virus infection. To clarify the role of mast cells in the development of clinical Dengue fever, we compared the plasma levels of several mast cell-derived mediators (vascular endothelial cell growth factor [VEGF], soluble VEGF receptors [sVEGFRs], tryptase, and chymase) and -related cytokines (IL-4, -9, and -17) between patients with differing severity of Dengue fever and healthy controls.

**Methodology/Principal Findings:**

The study was performed at Children's Hospital No. 2, Ho Chi Minh City, and Vinh Long Province Hospital, Vietnam from 2002 to 2005. Study patients included 103 with Dengue fever (DF), Dengue hemorrhagic fever (DHF), and Dengue shock syndrome (DSS), as diagnosed by the World Health Organization criteria. There were 189 healthy subjects, and 19 febrile illness patients of the same Kinh ethnicity. The levels of mast cell-derived mediators and -related cytokines in plasma were measured by ELISA. VEGF and sVEGFR-1 levels were significantly increased in DHF and DSS compared with those of DF and controls, whereas sVEGFR-2 levels were significantly decreased in DHF and DSS. Significant increases in tryptase and chymase levels, which were accompanied by high IL-9 and -17 concentrations, were detected in DHF and DSS patients. By day 4 of admission, VEGF, sVEGFRs, and proteases levels had returned to similar levels as DF and controls. *In-vitro* VEGF production by mast cells was examined in KU812 and HMC-1 cells, and was found to be highest when the cells were inoculated with Dengue virus and human Dengue virus-immune serum in the presence of IL-9.

**Conclusions:**

As mast cells are an important source of VEGF, tryptase, and chymase, our findings suggest that mast cell activation and mast cell-derived mediators participate in the development of DHF. The two proteases, particularly chymase, might serve as good predictive markers of Dengue disease severity.

## Author Summary

To clarify the involvement of mast cells in the development of severe Dengue diseases, plasma levels of mast cell-derived mediators, namely vascular endothelial cell growth factor (VEGF), tryptase, and chymase, were estimated in Dengue patients and control subjects in Vietnam. The levels of the mediators were significantly increased in Dengue hemorrhagic fever (DHF) and Dengue shock syndrome (DSS) patients compared with those of Dengue fever (DF) and control (febrile illness and healthy subjects) patients, and the soluble form of VEGF receptors (sVEGFR)-1 and -2 levels were significantly changed in the patients with severe disease. After 2–4 days of admission, the mediator levels had returned to similar levels as those of DF and control subjects. Furthermore, the levels of the Th17 cell-derived mast-cell activators IL-9 and -17 were increased in DHF and DSS. *In-vitro* production of VEGF in human mast cells was significantly enhanced in the presence of IL-9 when these cells were inoculated with Dengue virus in the presence of human Dengue virus-immune serum. As mast cells are an important source of VEGF, and tryptase and chymase are considered to be specific markers for mast cell activation, mast cells and mast cell-derived mediators might participate in the development of DHF/DSS.

## Introduction

Dengue virus infection is associated with disease, ranging from Dengue fever (DF) to Dengue hemorrhagic fever (DHF) and/or Dengue shock syndrome (DSS). As severe diseases typically develop in individuals suffering secondary Dengue virus infection, host immunological factors appear to play a role in DHF and DSS [1]. DHF and DSS are characterized by increased vascular permeability and hemorrhagic manifestations [2], with the former phenotype recognized as the hallmark of these severe forms of Dengue. However, the cellular factors and immune molecules underling the development of DHF and DSS are not well understood.

Recent studies on Dengue virus infection have demonstrated that the serum levels of vascular endothelial cell growth factor (VEGF)-A (formerly VEGF) are elevated in DHF patients [3]. VEGF/vascular permeability factor (VPF) was first identified and characterized as a potent stimulator of endothelial permeability [4], and was shown to increase vascular permeability 50,000 fold more efficiently than histamine [5]. VEGF was subsequently reported to promote the proliferation, migration, and survival of endothelial cells [6]. VEGF is a member of a growing family of related proteins that includes VEGF-B, -C, -D, and placental growth factor [7]. A potential candidate for the VEGF-binding molecule is the soluble form of its receptor. At least two types of VEGF receptors are expressed on endothelial cells; both are transmembrane receptor tyrosine kinases, namely, VEGFR-1 or Fms-like tyrosine kinase 1 (Flt-1), and VEGFR-2 or kinase insert domain receptor (KDR) [8]. VEGFR-1 is expressed on monocyte-macrophage lineages other than endothelial cells, whereas VEGFR-2 is expressed primarily on endothelial cells and their progenitors [9], [10]. In addition to its role in promoting endothelial permeability and proliferation, VEGF may contribute to inflammation and coagulation. For example, under *in-vitro* conditions, VEGF induces the expression of several types of cell adhesion molecules, including E-selectin, intercellular adhesion molecule 1 (ICAM-1), and vascular cell adhesion molecule 1 (VCAM-1), in endothelial cells and promotes the adhesion of leukocytes [11], [12]. Moreover, VEGF signaling up-regulates tissue factor mRNA expression, and protein and procoagulant activities [13]. The proinflammatory/procoagulant effects of VEGF are mediated, at least in part, by the activation of the transcription factors NF-κB, Egr-1, and NFAT. VEGF has been implicated as a pathophysiological mediator in several human disease states, including rheumatoid arthritis, cancer, and inflammatory bowel disease [14]–[16].

Dengue patients typically exhibit increased levels of urinary histamine, which is a major granule product of mast cells and whose levels correlate with disease severity [17]. A large autopsy study of 100 DHF cases from Thailand found that mast cells in connective tissue around the thymus exhibited swelling, cytoplasmic vacuolation, and loss of granule integrity, which are suggestive of mast cell activation [18]. Although recent *in-vitro* studies have also reported the involvement of mast cells in Dengue virus infection [19], [20], the potential role of mast cells in severe Dengue disease has not yet been explored.

The activation of mast cells, which reside mainly in tissues and are associated closely with blood vessels and nerves [21], [22], is tightly linked with local increases in vascular permeability in allergic disease. Mast cells are key effector cells in IgE-dependent immune responses, such as those involved in the pathogenesis of allergic disorders or in certain instances of immunity to parasites [23]. Recent works have revealed another aspect of mast cell effector function, and mast cells play important roles in inflammation and host defenses against foreign pathogens [24], [25].

Mast cells synthesize and release a range of biologically active substances, including proteases, biogenic amines, cytokines, chemokines, and lipid mediators [26]. Mast cell proteases are key protein components of secretory mast cell granules and are essential for innate antimicrobial inflammatory responses [27]–[29]. It is estimated that mast cell proteases account for >25% of total mast cell protein [30] and that human skin mast cells contain a total of ∼16 µg tryptase and chymase per 10^6^ cells [31]. Mast cell proteases, tryptase, and chymase are serine proteases with trypsin- or chymotrypsin-like substrate specificities, and are the major proteins stored and secreted by mast cells. Measurement of the serum (or plasma) levels of these proteases are recommended in the diagnostic evaluation of systemic anaphylaxis and mastocytosis, with total tryptase levels generally reflecting either the increased burden of mast cells in patients with all forms of systemic mastocytosis, or the decreased burden of mast cells associated with cytoreductive therapies in these disorders. Tryptase and chymase levels generally reflect the magnitude of mast cell activation and are typically elevated during systemic anaphylaxis. Secreted tryptase and chymase promote inflammation, matrix destruction, and tissue remodeling by several mechanisms, including the destruction of procoagulant, matrix, growth, and differentiation factors, and the activation of proteinase-activated receptors, urokinase, metalloproteinases, and angiotensin. In addition, these two serine proteases also modulate immune responses by hydrolyzing chemokines and cytokines, and can also suppress inflammation by inactivating allergens and neuropeptides responsible for inflammation and bronchoconstriction. Thus, similar to mast cells themselves, mast cell serine proteases play multiple roles in host defenses, which may be either beneficial or harmful depending on the specific conditions. As substantial levels of tryptase and chymase are only found in mast cells, these proteases are considered to be selective markers of mast cell activation [26]. The importance of cytokines and chemokines together with mast cells in the pathogenesis of Dengue virus infection has been demonstrated [19], [20], however, the roles of the mast cell-specific proteases, tryptase and chymase, remain unclear.

Here, to determine the roles of mast cells and mast cell-derived mediators in DHF and DSS, we first measured the levels of VEGF, soluble forms of VEGFR-1 and -2, tryptase, and chymase in the plasma of Dengue patients and healthy control subjects. Moreover, because IL-9 has been reported as a T cell-derived growth factor of mast cells [32]–[34] and more recently has been implicated as a Th17-derived cytokine that contributes to inflammatory diseases, the involvement of IL-9 and IL-17 in Dengue infection was also investigated.

## Methods

### Study population and Dengue classification

The study was performed at two hospitals, Children's Hospital No. 2 in Ho Chi Minh City (HCMC) and the Center for Preventive Medicine in Vinh Long Province (VL), Vietnam. The enrolment was a consecutive sequence of hospitalized children at each hospital. The inclusion criteria on admission to the hospital were age (6 months to 15 years old) and ethnicity (Kinh race). A total of 103 subjects from HCMC and VL were enrolled in this study during 2002–2005 (Table 1). The patients were suspected to have Dengue virus infection based on clinical symptoms at admission. After hospitalization, the patients were diagnosed using standardized serology techniques, as described below, and the WHO (1997) classification criteria for Dengue virus infection [35]. It was reported that the sensitivity of WHO criteria for DSS in Vietnam was only 82%, mainly due to the lack of evidence for thrombocytopenia [36]. Therefore, we basically followed the WHO criteria, but included patients lacking a significant reduction of platelet count, which accounted for no more than 11% of all DHF/DSS cases. Our classification scheme met the requirements of the simplified Integrated Management of Childhood Illness (IMCI) classification system, which is based on plasma leakage as a hallmark of severe dengue disease (DHF/DSS) [37].

**Table 1:** Characteristics of the Dengue patients.

Characteristic	DF (n = 19)	DHF (n = 43)	DSS (n = 41)
Sex (Male: Female)	12∶7	22∶21	21∶20
Fever (mean±SD °C)	37.9±0.7	39.2±0.7	39.1±0.8
Primary infection	9	11	10
Secondary infection	10	32	31
Dengue Virus 1	1	16	4
Dengue Virus 2	1	14	6
Dengue Virus 3	2	4	0
Dengue Virus 4	0	0	0
Dengue Virus (-)	15	9	31
Conjunctive bleeding	0	0	1
Subcutaneous bleeding	0	39	40
GI bleeding*	0	0	5
Plasma leakage signs**	0	0	11
Hematocrit	0	43	41
Thrombocytopenia	0	43	41
Hepatomegaly	0	11	32
Narrow blood pressure	0	0	23
Neurologic disorder	0	0	1
Shock	0	0	41

Dengue classification was performed according to the definitions of the World Health Organization (WHO) [27]. DHF classification required fever or a history of acute fever, bleeding manifestation, and signs of plasma leakage, which included hemoconcentration, ascites, or pleural effusion with evidence of thrombocytopenia. DSS classification required DHF manifestation plus evidence of clinical hypovolemic shock. Cases of Dengue were confirmed by Dengue virus RNA detection by RT-PCR and IgM antibody capture (MAC) ELISA in the first or second paired samples. Dengue virus was isolated using plasma samples collected between days 4–6. Primary or secondary infection was determined by the AFRIMS method. Hct increase was determined by a >20% increase compared with the normal range of the population. Thrombocytopenia was defined as <100,000 platelets/mm^3^.

*GI bleeding: Gastrointestinal bleeding.

**Plasma leakage signs: Ascites and pleural effusion.

Plasma samples were obtained from the 19 DF, 43 DHF, and 41 DSS patients on the day of admission, and an additional 189 plasma samples from healthy, unrelated school children living in HCMC and VL who had no symptoms of Dengue virus infection were collected as control samples. Eighteen (male: 12, female: 6) plasma samples were also collected from school children with a febrile illness (38.9±0.9°C: mean±SD) without an obvious source of infection, including Dengue virus.

This study was approved by the institutional ethical review committees of the Institute of Tropical Medicine, Nagasaki University, Jikei University School of Medicine in Tokyo, and the Pasteur Institute in Ho Chi Minh City. Written informed consent was obtained from the parents or legal guardians of the subjects upon enrollment.

### Sample collection and serological diagnosis

The sample collection and serological diagnosis performed in this cohort study were identical to those reported in our previous study [38]. Blood samples were collected from patients with suspected Dengue infection at the time of admission (day 0) and twice during the following four days (days 2 and 4). Plasma samples were used for the titration of anti-Dengue virus IgM and IgG antibodies, virus isolation, and RT-PCR for the determination of viral serotype. Dengue virus infection was determined by previously established serologic criteria for IgM/IgG ELISAs to Dengue virus (DEN 1–4) and Japanese encephalitis virus in paired plasma, collected with at least three-day intervals [39]. IgM and IgG ELISAs were performed using kits obtained from the Pasteur Institute, HCMC and were considered positive if the ratio of optical density (OD) of test sera to the OD of negative control plasma was ≥2.3 [35]. The cases were diagnosed as secondary infection when the DV IgM-to-IgG ratio was <1.8 [40].

Dengue virus serotyping was performed as previously reported [38]. Briefly, acute plasma samples were used to inoculate C6/36 (*Aedes albopictus*) cells, virus was obtained, and the Dengue virus serotype was then identified using either a direct or indirect fluorescent antibody technique with monoclonal antibodies supplied by the Centers for Disease Control and Prevention (Fort Collins, CO, USA) [41]. Viral RNA was also extracted from the acute plasma samples with the QIAamp Viral RNA Mini Kit (Qiagen, Hilden, Germany) for the molecular detection of Dengue virus and confirmation of its serotype, as previously described [41]. Briefly, cDNA from the Dengue virus genome RNA was amplified with the Ready-to-go RT-PCR test kit (Amersham, MA, USA) using a consensus primer set (D1 and D2) [31]. The serotype was then determined by semi-nested PCR using specific primer sets (TS1, TS2, TS3, and TS4) to amplify serotype-specific fragments from the regions encoding the capsid and membrane proteins of Dengue virus [39].

### ELISA assay

The plasma levels of VEGF (VEGF-A), sVEGFR-1, sVEGFR-2, IL-9, and IL-17 in samples from Dengue patient (DF, DHF, and DSS) and control groups (febrile illness and healthy subjects) were measured by ELISA kits (R&D Systems, Minneapolis, MN, or Peprotech Inc., Rocky Hill, NJ). The levels of tryptase or chymase in plasma from the Dengue patients and control groups, and the culture supernatants of mast cells were examined by ELISA kits (CSB, Newark, ED or Otsuka Pharmaceutical Co., Tokushima, Japan).

### Dengue virus infection and in-vitro production of VEGF

The human mast cell/basophil line KU812 [42] and human mast cell line HMC-1 [43] were maintained in RPMI 1640 medium or IMDM (Invitrogen, Grand Island, NY). In the infection experiments, Dengue virus 2 (DV16681 strain) was propagated in the C6/36 cell line, and virus titers were then determined by plaque assay using BHK-21 cells [44]. In control experiments, the virus was rendered nonreplicative by placing a sample aliquot under a germicidal lamp (125 mJ/10 min, UV irradiation at 254 nm) at a distance of 5–6 cm, followed by a 30-min incubation on ice [44]. For infection, HMC-1 or KU812 cell pellets were adsorbed at 4°C for 90 min with aliquots of Dengue virus or UV-inactivated virus, Dengue virus in combination with human dengue virus immune serum (1∶1,000 or 1∶10,000 final dilution), UV-inactivated virus in combination with human dengue virus immune serum (1∶1,000 or 1∶10,000 final dilution), or Dengue virus in combination with normal human serum (1∶1,000 final dilution) (premixed at 4°C for 90 min). Dengue virus 2 convalescent-phase sera were used in the antibody-dependent enhancement of Dengue virus infection. Mast cells were infected at a multiplicity of infection (MOI) of 3 plaque forming units (pfu)/cell. Following adsorption, cells were washed and plated in 96-well plates (0.25 mL/well) at 1×10^6^ cells/mL and then incubated at 37°C in 5% CO_2_ for 24 h. To examine the effects of IL-9 on the production of VEGF from mast cells, mast cells treated with Dengue virus and antibody were incubated with or without recombinant human IL-9 (200 ng/mL, Peprotech, Rocky Hill, NJ). Activation of mast cells with Compound (C) 48/80 (300 µg/ml, Sigma-Aldrich, St. Louis, MO) was used as a positive control, and the culture supernatant of C6/36 cells was used as a negative control. For the measurement of VEGF levels in culture supernatants, culture supernatants were collected from each well after incubation and then stored at −80°C until being subjected to ELISA.

### Fluorescence microscopy

KU812 cells were inoculated with Dengue virus (MOI, 3) or Dengue virus-antiserum combinations. After incubation for 24 h, cells were fixed with 4% paraformaldehyde, washed, and then permeabilized with 0.1% saponin for 1 h at room temperature. Samples were then washed and incubated with mouse anti-Dengue virus monoclonal antibody 1B7 [45] and isotype-matched mouse IgG2a antibody (negative control, R&D Systems) on ice for 1 h, which were employed as primary antibodies. Subsequently, samples were washed and incubated with FITC-labeled anti-mouse IgG antibody (R&D Systems) for 1 h on ice. Cytospins were made for each sample, and positive cells were observed by fluorescence microscopy.

### Statistical analysis

Plasma VEGF, sVEGFRs, IL-9, IL-17, tryptase, and chymase levels were compared between the Dengue (DF, DHF, or DSS) and control groups (febrile illness and healthy subjects) using the unpaired Student's t test. VEGF levels in the *in-vitro* experiments were also compared between the Dengue virus infection and control (UV-inactivated Dengue virus and Medium alone) samples using the unpaired Student's t test. A value of p<0.05 was considered statistically significant.

## Results

### VEGF and sVEGFR levels in Dengue patients

**Figure 1:**
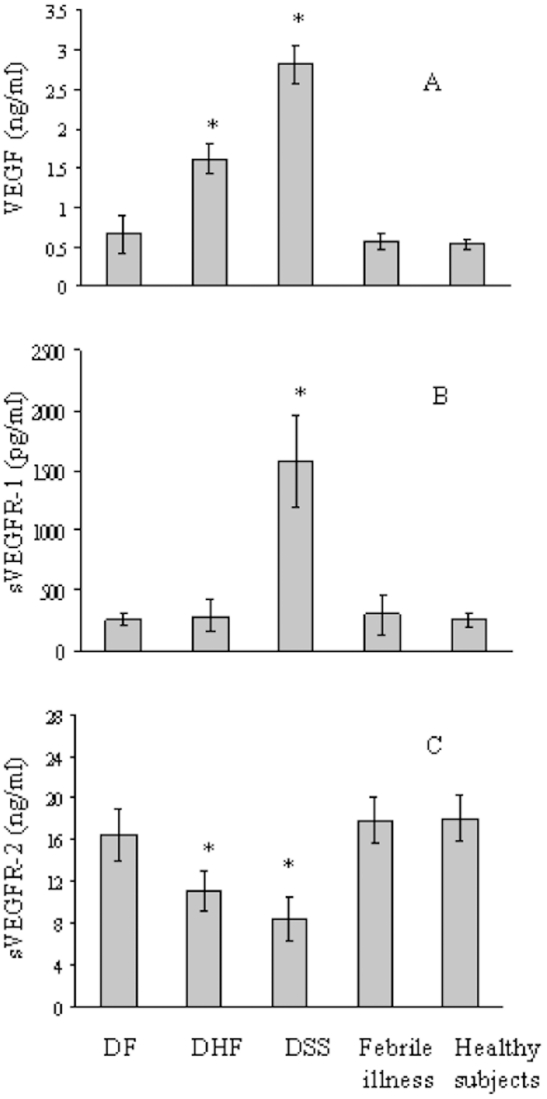
Plasma levels of VEGF, and sVEGFR-1 and -2 in Dengue patients and control groups. Plasma levels of VEGF (A), and sVEGFR-1 (B) and -2 (C) were examined for Dengue patients (DF, DHF, and DSS) on admission and for control subjects (febrile illness and healthy subjects) by ELISA. VEGF *p<0.01 (DHF and DSS versus DF, febrile illness or healthy subjects), sVEGFR-1 *p<0.01 (DSS versus DF, DHF, febrile illness or healthy subjects), sVEGFR-2 *p<0.01 (DHF and DSS versus DF, febrile illness or healthy subjects). Representative results from three independent experiments are shown.

As mast cells are an important source of VEGF [46], [47], we first measured VEGF levels in plasma samples from the DF (n = 19), DHF (n = 43), and DSS (n = 41) patient groups, and the control group, which consisted of febrile illness and healthy subjects. On day 0 (admission), the VEGF plasma levels were significantly higher in DHF and DSS than those in DF, and febrile illness and healthy subjects (Fig. 1A). The sVEGFR-1 levels in plasma were higher in DSS than those in DF, DHF, febrile illness and healthy subjects (Fig. 1B). In contrast with sVEGR-1, the levels of sVEGFR-2 were dramatically decreased in DHF and DSS compared with DF or febrile illness and healthy subjects (Fig. 1C).

**Figure 2:**
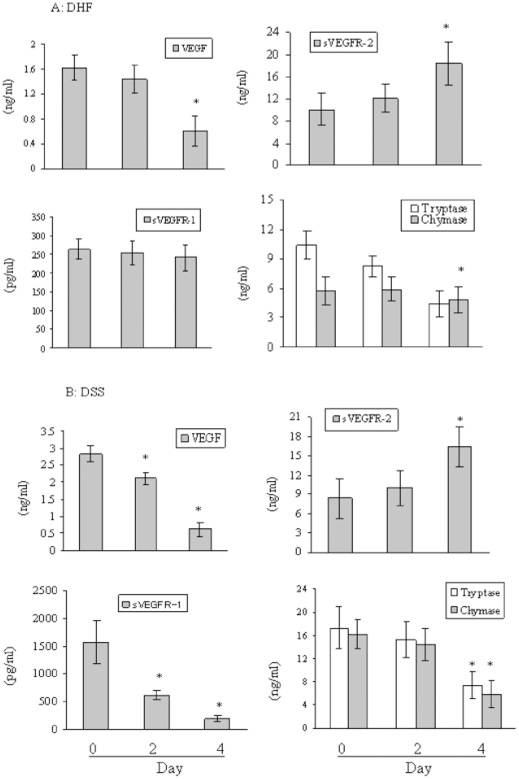
Plasma levels of VEGF, sVEGFRs, tryptase, and chymase in Dengue patients during the admission period. Plasma levels of VEGF, sVEGFR-1 and -2, tryptase, and chymase were examined for Dengue patients with DHF (A) and DSS (B) by ELISA. VEGF in DHF *p<0.01 (day 4 versus day 0 and 2) and DSS *p<0.01 (day 2 and 4 versus day 0), sVEGFR-1 in DSS *p<0.01 (day 2 and 4 versus day 0), sVEGFR-2 in DHF *p<0.01 (day 4 versus day 0 and 2) and DSS *p<0.01 (day 4 versus day 0 and 2), tryptase in DHF *p<0.01 (day 4 versus day 0 and 2) and DSS *p<0.01 (day 4 versus day 0 and 2), chymase in DSS *p<0.01 (day 4 versus day 0 and 2). Results are representative of two independent experiments.

We next examined the levels of VEGF and sVEGFRs in DHF (n = 21) and DSS (n = 27) patients during the admission period (Fig. 2). The VEGF levels in DHF and DSS, and sVEGFR-1 levels in DSS were significantly higher than those of DF or healthy controls on the day of admission (day 0); however, 2–4 days later (convalescence), their levels had gradually declined to comparable levels with DF, febrile illness, and healthy subjects by the convalescent phase (day 4; VEGF DF: 0.61±+0.24 ng/ml, febrile illness: 0.57±0.11 ng/ml, and healthy subjects: 0.52±0.17 ng/ml; sVEGFR-1 DF: 180.9+55.3 pg/ml, DHF: 223.6±136 pg/ml, febrile illness: 201.3±167.1 pg/ml, and healthy subjects: 195.1±59.1 pg/ml). The plasma levels of sVEGFR-2 in DHF and DSS patients were significantly lower compared to those of DF, febrile illness, and healthy subjects; however, the levels were comparable between these groups by day 4 (Fig. 2). Taken together, these findings suggested the possibility that VEGF and sVEGFRs participated in severe Dengue virus infection.

### Tryptase and chymase levels in Dengue patients

**Figure 3:**
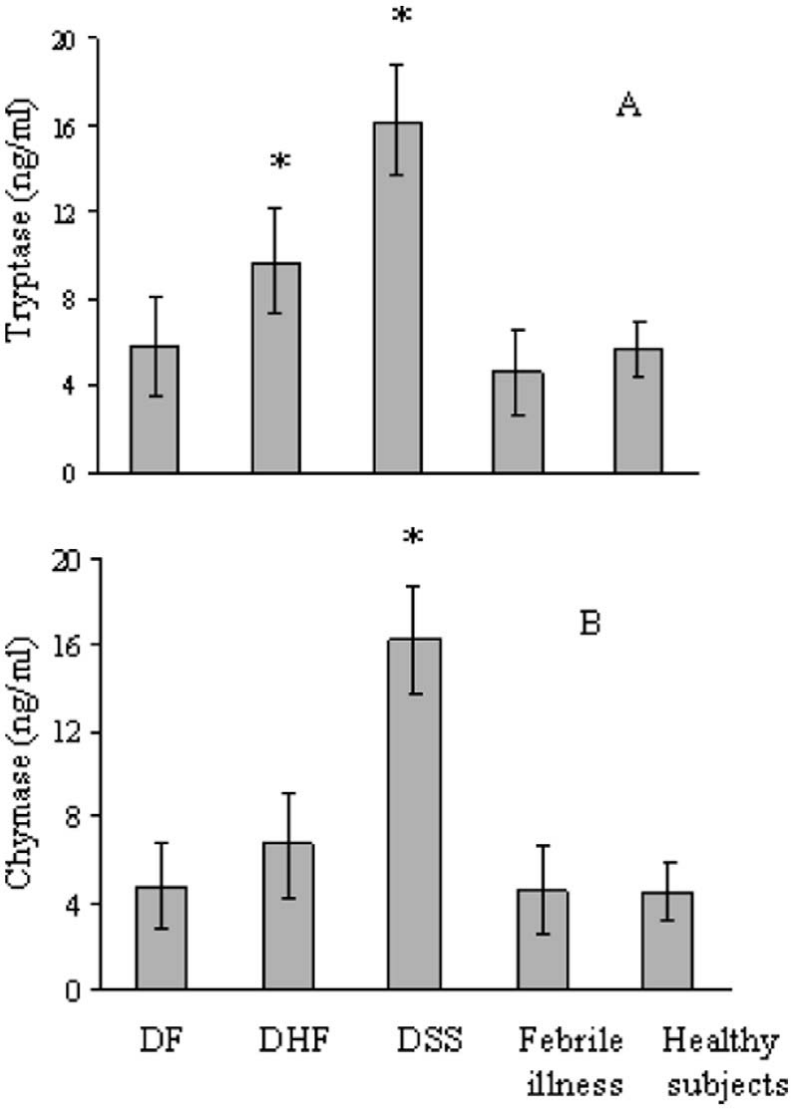
Plasma levels of tryptase and chymase in Dengue patients and control groups. Plasma levels of tryptase (A) and chymase (B) were examined for Dengue patients (DF, DHF, and DSS) on admission and for control groups (febrile illness and healthy subjects) by ELISA. Tryptase *p<0.01 (DHF and DSS versus DF, febrile illness or healthy subjects), and Chymase *p<0.01 (DSS versus DF, DHF, febrile illness or healthy subjects). Representative results from three independent experiments are shown.

We also measured the tryptase and chymase levels in plasma collected from the Dengue patients (day 0) and controls by ELISA. Plasma tryptase levels increased significantly in DHF and DSS compared with DF, febrile illness, and healthy subjects (Fig. 3). In contrast, the chymase levels were increased significantly in DSS compared with DF, DHF, febrile illness, and healthy subjects (Fig. 3). We next measured the plasma levels of tryptase and chymase in DHF (n = 21) and DSS (n = 27) patients during the admission period and found that the protease levels had gradually declined by days 2 and 4 to a comparable level with those of DF, febrile illness, and healthy subjects (chymase DF: 4.8±2 ng/ml, DHF: 6.7±2.4 ng/ml, febrile illness: 6.2±2 ng/ml, healthy subjects: 4.5±1.3 ng/ml, tryptase DF: 7.4±4.3 ng/ml, febrile illness: 6.7±2.6 ng/ml, healthy subjects: 7.7±3.4 ng/ml) (Fig. 2). These results suggested that mast cells and mast cell-derived proteases participated in the severe form of Dengue virus infection.

### IL-4, IL-9, and IL-17 levels in Dengue patients

**Figure 4:**
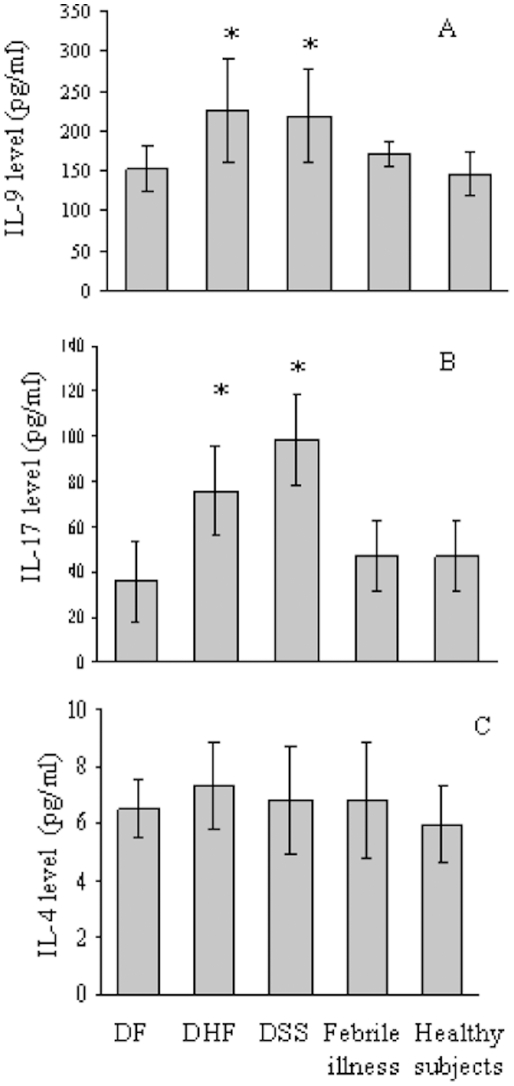
Plasma levels of IL-9, -17, and -4 in Dengue patients and control groups. Plasma levels of IL-9 (A), -17 (B), and -4 (C) were examined for Dengue patients (DF, DHF and DSS) and control groups (febrile illness and healthy subjects) by ELISA. IL-9, *p<0.01 (DHF and DSS versus DF, febrile illness and healthy subjects), IL-17, *p<0.01 (DHF and DSS versus DF, febrile illness and healthy subjects). Representative results from three independent experiments are shown.

As IL-9 has been reported as a T cell-derived mast cell growth factor [32]–[34] and more recently, is implicated as a Th17-derived cytokine that can contribute to inflammatory diseases, we investigated the involvement of IL-9 and IL-17 in Dengue virus infection. The levels of IL-9 and IL-17 in Dengue patients on day 0, and those in blood samples collected from febrile illness and healthy subjects were measured by ELISA. The analysis showed that IL-9 and IL-17 levels were significantly increased in DHF and DSS compared with those in DF, febrile illness, and healthy subjects (Figs. 4A and B).

Although these results suggested that IL-9 and IL-17 participate in Dengue virus infection, IL-9 may act additively or synergistically with other factors, such as other Th2 cytokines, to induce optimal mast cell responses. To examine the possibility that Th2 cytokines affect mast cell responses in Dengue virus infection, IL-4 levels were also examined in plasma from Dengue patients and control groups (Fig. 4C). We found comparable levels of IL-4 between Dengue patients and control groups, suggesting the involvement of IL-9 and -17 in Dengue virus infection.

### *In-vitro* production of VEGF in mast cells

To investigate if Dengue virus induces VEGF production from mast cells, the *in-vitro* production of VEGF in the human mast cell lines KU812 and HMC-1 was examined. KU812 and HMC-1 cells were inoculated with Dengue virus in the presence of either human Dengue virus-immune or normal human serum, and VEGF levels in the culture medium were assessed 24 h after viral inoculation. As the antibody-dependent enhancement of infection in KU812 and HMC-1 cells was observed at 1∶1,000 and 1∶10,000 dilutions of human Dengue virus-immune serum in preliminary experiments (data not shown), a 1∶1,000 dilution was used in the *in-vitro* experiments in this study.

The production of VEGF was observed in both KU812 and HMC-1 cells after exposure to Dengue virus in the presence human Dengue virus-immune serum, however, VEGF levels were higher in KU812 cells (Table 2). No significant increase of VEGF level was observed when Dengue virus was inoculated with normal human serum (1∶1,000 final dilution) or when UV-inactivated Dengue virus was inoculated with human Dengue virus immune or normal human serum. In addition, no VEGF production by KU812 and HMC-1 cells was observed after mock-infection with human Dengue immune or normal human serum. These results suggested the importance of antibody to Dengue virus for mast cell secretion of VEGF *in vitro*.

**Table 2:** VEGF production by KU812 and HMC-1 cells exposed to Dengue virus.

Cell line	Serum	DV	UDV	C3/36^a^	C48/80^b^ (ng/ml)
KU812	HDIS	4.2±0.9*	0.4±0.3	0.4±0.1	7.5±0.8*
KU812	NHS	0.5±0.2	0.4±0.2	0.4±0.1	7.3±0.4*
HMC-1	HDIS	2.3±0.4*	0.5±0.2	0.4±0.1	4.6±0.4*
HMC-1	NHS	0.5±0.1	0.4±0.1	0.3±0.2	4.2±0.7*

KU812 and HMC-1 cells were inoculated with Dengue virus-2 (DV) or UV-irradiated Dengue virus-2 (UDV) in the presence of human Dengue-immune serum (HDIS, 1∶1000 final dilution) or normal human serum (NHS, 1∶1000 final dilution), and VEGF levels in culture supernatants were then examined 24 h later. Significant VEGF production was not observed when KU812 and HMC-1 cells were infected with DV alone (data not shown).

HDIS in KU812*p<0.01 (HDIS and C48/80 versus UDV or C3/36), NHS in KU812 *p<0.01 (C48/80 versus DV, UDV or C3/36), HDIS in HMC-1 *p<0.01 (DV versus UDV, C3/36 or C48/80), NHS in HMC-1 *p<0.01 (C48/80 versus DV, UDV, or C3/36).

a : C3/36 medium alone served as a negative control.

b : Activation of mast cells with C48/80 (300 µg/ml) was used as a positive control.

**Figure 5:**
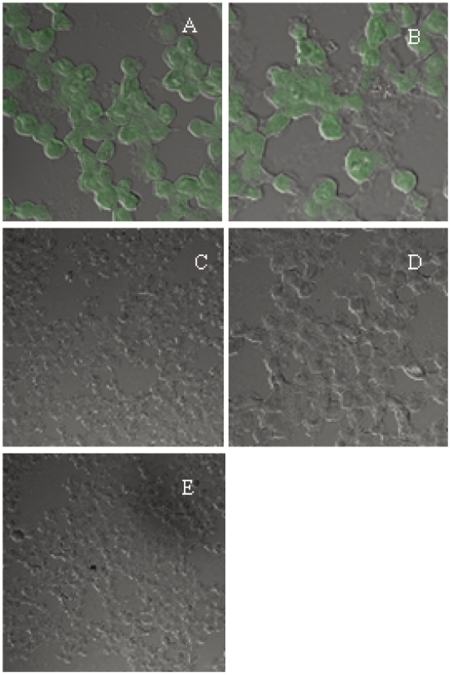
Immunofluorescence staining of Dengue virus-infected KU812 cells. KU812 cells inoculated with a combination of Dengue virus-2 and human Dengue virus-immune or normal human serum were harvested 24 h post-infection. The harvested cells were incubated with mouse anti-Dengue virus monoclonal antibody 1B7 (30) or isotype-matched mouse IgG2a antibody (negative control) as a primary antibody. Anti-mouse IgG labeled with FITC was used as a secondary antibody. (A)–(C) KU812 cells inoculated with a combination of Dengue virus and human Dengue virus-immune serum (A 1∶1000, ×40; B 1∶10000 final dilution, ×40) or normal human serum (C 1∶1000 final dilution, ×20). (D)–(E) KU812 cells inoculated with a combination of UV-inactivated Dengue virus and human Dengue virus-immune serum (D 1∶1000, ×40) or human Dengue immune serum alone (E 1∶1000, ×20). Similar results were obtained from two additional experiments.

As it is known that KU812 and HMC-1 cells are permissive to Dengue virus infection when the virus is inoculated together with human Dengue immune serum [20], the antibody-dependent infection of KU812 cells with Dengue virus was examined by immunofluorescence analysis in the presence and absence of human Dengue immune serum 24 h after the inoculation. Positive immunofluorescence was only observed in cells infected in the presence of human Dengue virus-immune serum, suggesting the occurrence of permissive infection of Dengue virus (Fig. 5). To determine the role of IL-9 in VEGF production by mast cells, KU-812 and HMC-1 cells were inoculated with Dengue virus and human Dengue virus-immune serum (1∶1,000 final dilution) in the presence and absence of IL-9. Although a low level of VEGF production by KU-812 and HMC-1 cells was observed without IL-9, VEGF levels were significantly increased in the presence of IL-9 (Table 3). The effect of IL-9 on VEGF production by KU812 and HMC-1 cells was not observed in the presence of normal human serum (data not shown). Taken together, these findings suggested the possibility that Dengue virus induces VEGF secretion from human mast cells during infection, and that IL-9 supports the production of VEGF in mast cells.

**Table 3:** Effect of IL-9 on VEGF production in KU812 and HMC-1 cells.

Cell line	IL-9	DV	UDV	C3/36^a^	C48/80^b^ (ng/ml)
KU812	+	6.4±1.4*	0.5±0.3	0.6±0.3	8.4±1. 2*
KU812	−	4.1±1.5*	0.4±0.1	0.4±0.2	7.4±1.8*
HMC-1	+	3.5±0.4*	0.6±0.1	0.5±0.2	4.9±0.4*
HMC-1	−	2.3±0.2*	0.4±0.2	0.3±0.1	4.2±0.2*

KU812 and HMC-1 cells were inoculated with Dengue virus-2 (DV) or UV-irradiated Dengue virus-2 (UDV) in the presence or absence of IL-9 (200 ng/ml), and VEGF levels were then examined. VEGF production by KU812 and HMC-1 cells was significantly increased in the presence of IL-9 when the cells were cultured with DV and human Dengue virus-immune serum (1∶1000 final dilution). However, significant VEGF production was not observed in cells when KU812 and HMC-1 cells were infected with DV or UDV in the presence of normal human serum (1∶1000 final dilution) and IL-9 (data not shown). Significant VEGF production was not observed when KU812 and HMC-1 cells were infected with DV alone (data not shown).

IL-9+ in KU812 *p<0.01 (DV and C48/80 versus UDV or C3/36), IL-9− in KU812 *p<0.01 (DV and C48/80 versus UDV or C3/36), IL-9+ in HMC-1 *p<0.01 (DV and C48/80 versus UDV or C3/36), IL-9− in HMC-1 *p<0.01 (DV and C48/80 versus UDV or C3/36).

a : C3/36 medium alone (mock infection) served as a negative control.

b : Activation of mast cells with C48/80 (300 µg/ml) was used as a positive control.

*p<0.01.

## Discussion

Recently, Srikiatkhachorn et al. [48] compared the plasma levels of VEGF-A and sVEFGR-1 and -2 between DHF and DF patients, and found a rise of VEGF-A and decline of sVEGFR-2 levels in DHF patients, with the severity of plasma leakage inversely correlating with sVEGFR-2 levels. These findings seemed to be consistent with our present results that VEGF and sVEGFR-2 were significantly increased and reduced, respectively, in DHF and DSS patients. Although the reason why sVEGFR-2 levels are decreased in DHF and DSS patients is not clear, as VEGF binding to VEGFR-2 on endothelial cells results in receptor phosphorylation, changes in endothelial cell morphology and proliferation, and maintenance of physiological condition of blood vessels, decreased sVEGFR-2 levels in severe Dengue patients might represent the dysfunction of homeostasis in vascular endothelial cells and correlate with increased plasma leakage [49]. We additionally observed a significant increase of sVEGFR-1 levels in DSS patients, which suggests that activation of monocytes/macrophages by Dengue virus leads to increased expression of soluble and surface VEGFR-1 on cells during severe Dengue infection, as was previously reported [49].

Regarding the relationship between VEGF level and severity of Dengue virus infection, Tseng et al. [3] observed the elevation of circulating VEGF levels in adult DHF patients during the early phases of Dengue infection, as compared to DF patients and healthy controls. In a study of a pediatric population with DHF, Srikiatkhachorn et al. [6] also detected a rise in circulating VEGF in the early febrile and defervescent stages of Dengue infection, but not during the later convalescent stage. However, subsequent studies reported contradictory findings, as increased circulating VEGF concentrations were not observed during the early febrile and toxic stages in DHF, but lower VEGF concentrations were detected in patients with more severe Dengue infection [50]–[52]. Several underlying reasons may explain these differences, such as poor study design, small sample size, and the lack of a standardized collection methodology and storage of blood samples used for the measurement of VEGF. In addition, VEGF is also expressed at low levels in a wide variety of normal adult human and animal tissues, and at higher levels in a few selected sites, namely, podocytes of the renal glomerulus, cardiac myocytes, prostatic epithelium and semen, and certain epithelial cells of the adrenal cortex and lung [53]. Dovrak et al. [54] reported that VEGF is substantially overexpressed at both the mRNA and protein levels in a high percentage of malignant animal and human tumors, as well as in many transformed cell lines. Thus, studies of VEGF production by mast cells during Dengue virus infection are complicated by these alternate sources of VEGF in human and animals, and may affect circulating VEGF levels.

Incubation of KU812 and HMC-1 cells with Dengue virus in the presence of human Dengue virus-immune serum resulted in enhanced VEGF production, which was not observed when UV-inactivated Dengue virus was incubated with human Dengue virus-immune serum or when Dengue virus was used alone to infect KU812 cells (Table 2). As the permissive infection of Dengue virus was observed in KU812 cells (Fig. 5), these findings suggest the critical importance of antibodies to Dengue virus for VEGF production by highly infected mast cells and indicate that infected mast cells can secrete VEGF without stimulation through FcεRI. Our results appear consistent with the findings that Dengue virus infection induces the production of chemokines by human mast cells without stimulation of FcεRI in the presence of human Dengue immune serum [22]. Brown et al. [55] reported that FcγRII plays a dominant role in antibody-enhanced Dengue virus infection of the human mast cell lines HMC-1 and KU812, and in the associated CCL5 release. In studies of DHF epidemics, Halstead et al. [56] and Guzman et al. [57] demonstrated that secondary infection is the most important host risk factor for DHF.

Boesiger et al. and Grützkau et al. [46], [47] reported that mouse and human mast cells produce and secrete VPF/VEGF, and release VEGF upon stimulation through FcεRI or after challenge with chemical mast cell activators. Notably, the FcεRI-dependent secretion of VEGF by either mouse or human mast cells is significantly increased in cells that have undergone upregulation of FcεRI surface expression by preincubation with IgE. As Koraka [58] reported that Dengue virus-specific IgE levels were significantly higher in DHF and DSS patients compared to those in DF and non-Dengue patients, FcεRI may be important for mast cell activation via IgE antibody in Dengue virus infection. However, we did not determine whether the patient sera collected in the present contained IgE antibody against Dengue virus. To clarify the importance of FcεRI for VEGF production by mast cells in Dengue virus infection, further studies are needed.

High levels of VEGF in culture supernatants were detected when KU812 and HMC-1 cells were cultured in the presence of IL-9 (Table 3). As IL-9 enhances the survival of mast cells and induces their production of proinflammatory cytokines, including Th1 and Th2 cytokines [59], it is possible that IL-9 primes HMC-1 and KU812 cells *in vitro* to respond to Dengue virus infection by promoting VEGF production. To evaluate the contribution of IL-9 and IL-17 to Dengue virus infection, we measured the plasma levels of these two cytokines in Dengue patients and found that both IL-9 and IL-17 were significantly increased in DHF and DSS compared with DF, febrile illness and healthy subjects. These findings suggest that Th9 and Th17 cells contribute to the inflammatory response to severe Dengue virus infection. It is possible that IL-9 may act additively or synergistically with other factors, such as additional Th2 cytokines, to induce the mast cell response observed in this study. However, as the level of IL-4 was not increased in the plasma of Dengue patients, our findings suggest the independent involvement of IL-9 secreted by Th2 cells in Dengue virus infection. Recently, IL-9-producing cells have been described as a new subset of the T helper cell population separate from Th2 that produces IL-9 in large quantities and contributes uniquely to immune responses [60], [61]. This cell population has been named ‘Th9’, and IL-9 secreted by T cells, particularly Th9 cells, may regulate chronic allergic inflammation [62]. Moreover, IL-9 has been recently proposed to function as a Th17-derived cytokine that contributes to inflammatory diseases [36].

Tryptase and chymase levels were significantly increased in DHF and DSS, and DSS, respectively, on admission compared with DF, febrile illness, and healthy subjects (Fig. 3). However, 2–4 days after admission, the levels of these proteases had returned to similar levels with the other patient groups (Fig. 2). These findings support the concept that mast cells and mast cell degranulation play important roles in the pathogenesis of DHF/DSS and might be suitable targets for new therapies and prevention of Dengue infection. However, it is presently unclear whether Dengue virus infection in mast cells directly induces chymase and tryptase production and secretion. Recently, Kitamura-Inenaga et al. [63] reported that encephalomyocarditis virus infection results in mast cell chymase and tryptase production *in vivo*, and additionally, viral infections have been shown to cause the accumulation of mast cells in the nasal mucosa during the first days of a symptomatic, naturally acquired respiratory infection [64]. However, the relevance and underlying mechanisms of mast cell infection and activation in the setting of viral infections remain to be characterized in detail.

Immunocytohistochemical studies in human tissues have identified two mast cell phenotypes distinguishable by their neutral protease content, namely the ‘mast cell-tryptase’ (MCT) phenotype and the ‘mast cell-tryptase-chymase’ (MCTC) phenotype [65]. MCT appears to be associated with immune system-related mast cells that play a primary role in host defenses and are preferentially located at mucosal surfaces. MCT mast cells are increased in number in areas of T lymphocyte infiltration and in allergic disease, and are reduced in number in acquired and chronic immunodeficiency syndromes [65]. In contrast, the MCTC phenotype appears to be associated with non-immune system-related mast cells that primarily function in angiogenesis and tissue remodeling, rather than immunologic protection, and are found predominantly in submucosal and connective tissues. In addition, MCTC mast cells are not increased in numbers in areas of heavy lymphocytic infiltration and are not decreased in number in immunodeficiency syndromes [65]. In the present study, a significant increase of chymase was observed in the plasma of DSS patients as compared with those of DF, DHF, and the control group, suggesting the possibility that MCTC mast cells contribute to the pathogenesis of severe forms of Dengue virus infection. However, further study is needed to clarify the roles of tryptase and chymase in severe Dengue virus infection.

Concerning the ability of mediators produced by mast cells other than VEGF to activate endothelial cells, King et al. [22] reported that Dengue virus plus Dengue virus-specific antibody treatment results in selective production of the T-cell chemoattractants RANTES, MIP-1α, and MIP-1β by KU812 and HMC-1 human mast cell-basophil lines. In addition, Brown et al. [66] demonstrated that antibody-enhanced Dengue virus infection of primary human cord blood-derived mast cells (CBMCs) and HMC-1 cells results in the release of ICAM-1 and VCAM-1, which subsequently activate human endothelial cells. St. John et al. [67] reported that the response to mast cell activation involves the *de novo* transcription of cytokines, including TNF-α and IFN-α, and chemokines, such as CCL5, CXCL12, and CX3CL1, which are well characterized to recruit immune effector cells, including cytotoxic lymphocytes, to sites of peripheral inflammation.

In conclusion, we found that mast cells and mast cell-derived mediators, namely VEGF, and the mast cell-specific proteases tryptase and chymase participate in the development of severe forms of Dengue virus infection, which is accompanied by elevated circulating levels of IL-9 and -17. As tryptase and chymase are known as selective markers of non-immune system-related activation of mast cells in submucosal and connective tissues, these two proteases, particularly chymase, might serve as good predictive markers of Dengue disease severity.
